# Molecular epidemiology of SARS-CoV-2 in Northern South Africa: wastewater surveillance from January 2021 to May 2022

**DOI:** 10.3389/fpubh.2023.1309869

**Published:** 2023-12-19

**Authors:** Lisa Arrah Mbang Tambe, Phindulo Mathobo, Nontokozo D. Matume, Mukhethwa Munzhedzi, Joshua Nosa Edokpayi, Amsha Viraragavan, Brigitte Glanzmann, Denis M. Tebit, Lufuno Grace Mavhandu-Ramarumo, Renee Street, Rabia Johnson, Craig Kinnear, Pascal Obong Bessong

**Affiliations:** ^1^HIV/AIDS & Global Health Research Programme, University of Venda, Thohoyandou, South Africa; ^2^Department of Biochemistry and Microbiology, University of Venda, Thohoyandou, South Africa; ^3^Discipline of Genetics, School of Life Sciences, University of KwaZulu-Natal, Pietermaritzburg, South Africa; ^4^Water and Environmental Management Research Group, University of Venda, Thohoyandou, South Africa; ^5^South African Medical Research Council Genomics Platform, Tygerberg, South Africa; ^6^Global Biomed Laboratories Inc., Lynchburg, VA, United States; ^7^Environment and Health Research Unit, South African Medical Research Council, Johannesburg, South Africa; ^8^Biomedical Research and Innovation Platform, South African Medical Research Council, Cape Town, South Africa; ^9^Division of Medical Physiology, Faculty of Medicine and Health Sciences, Centre for Cardiometabolic Research in Africa, Stellenbosch University, Stellenbosch, South Africa; ^10^Center for Global Health Equity, School of Medicine, University of Virginia, Charlottesville, VA, United States; ^11^School of Health Sciences, University of KwaZulu-Natal, Durban, South Africa

**Keywords:** SARS-CoV-2, WBE genomic surveillance, viral evolution, genetic characterization, RBD mutation analysis

## Abstract

**Introduction:**

Wastewater-based genomic surveillance of severe acute respiratory syndrome coronavirus 2 (SARS-CoV-2) provides a comprehensive approach to characterize evolutionary patterns and distribution of viral types in a population. This study documents the molecular epidemiology of SARS-CoV-2, in Northern South Africa, from January 2021 to May 2022.

**Methodology:**

A total of 487 wastewater samples were collected from the influent of eight wastewater treatment facilities and tested for SARS-CoV-2 RNA using quantitative reverse transcriptase polymerase chain reaction (qRT-PCR). SARS-CoV-2 positive samples with genome copies/mL ≥1,500 were subjected to allele-specific genotyping (ASG) targeting the Spike protein; 75 SARS-CoV-2 positive samples were subjected to whole genome sequencing (WGS) on the ATOPlex platform. Variants of concern (VoC) and lineages were assigned using the Nextclade and PangoLIN Software. Concordance for VoC between ASG and WGS analyses was determined. Sequence relationship was determined by phylogenetic analysis.

**Results:**

Seventy-five percent (365/487) of the influent samples were positive for SARS-CoV-2 RNA. Delta and Omicron VoC were more predominant at a prevalence of 45 and 32%, respectively, and they were detected as early as January and February 2021, while Beta VoC was least detected at a prevalence of 5%. A total of 11/60 (18%) sequences were assigned lineages and clades only, but not a specific VoC name. Phylogenetic analysis was used to investigate the relationship of these sequences to other study sequences, and further characterize them. Concordance in variant assignment between ASG and WGS was seen in 51.2% of the study sequences. There was more intra-variant diversity among Beta VoC sequences; mutation E484K was absent. Three previously undescribed mutations (A361S, V327I, D427Y) were seen in Delta VoC.

**Discussion and Conclusion:**

The detection of Delta and Omicron VoCs in study sites earlier in the outbreak than has been reported in other regions of South Africa highlights the importance of population-based approaches over individual sample-based approaches in genomic surveillance. Inclusion of non-Spike protein targets could improve the specificity of ASG, since all VoCs share similar Spike protein mutations. Finally, continuous molecular epidemiology with the application of sensitive technologies such as next generation sequencing (NGS) is necessary for the documentation of mutations whose implications when further investigated could enhance diagnostics, and vaccine development efforts.

## 1 Introduction

The coronavirus disease (COVID-19), caused by the severe acute respiratory syndrome coronavirus type 2 (SARS-CoV-2) is an acute respiratory infection (ARI) that has ravaged the world, causing over 696 million infections, with a mortality of over 6.9 million, as of October 2023 (1). South Africa alone has recorded over 4.07 million infections, and well over 102,000 deaths, as of October 2023. Throughout the pandemic, molecular epidemiology studies have been instrumental in providing information about viral genome organization, and mutational profiles, as well as the development of drug targets for treatment and vaccines to decrease mortality in those infected with SARS-CoV-2. This has been achieved mainly through whole genome sequencing (WGS), since it reveals critical epidemiological information (2) for virus classification, tracking global lineage transmission, and monitoring viral evolution (3, 4).

Over the course of 3 years of the pandemic, SARS-CoV-2 has evolved rapidly, due to its high mutation rate, estimated to be between 10^−5^ and 10^−3^ (5) that significantly impacts viral protein structures, function, and immunogenic characteristics (6, 7). These characteristics are strongly associated with the immunological response and clinical outcome in humans. The Spike protein (S-protein) of the virus functions mainly in binding to human cellular entry receptors (angiotensin-converting enzyme 2 – ACE2), which allows infection (8). Since the beginning of the COVID-19 pandemic, mutations detected in the S-protein have been used to characterize variants of concern (VoCs) and variants of interest (VOI) that arose over time. Both VoCs and VOIs are classified based on their potential impact, with VoCs regarded as posing the highest risk on the population. The WHO has classified five VoCs, which include: Alpha, Beta, Gamma, Delta, and Omicron (9).

By May 2020, the D614G mutation was widely reported to have overtaken the original Wuhan strain, and was observed in over 78% of clinical samples worldwide (10). As the pandemic progressed, specific key mutations developed in the S-protein of the virus, which led to increased infectivity and transmissibility. Mutations N501Y, DelH69V70, and P681H developed next, and were then classified as the Alpha variant (B.1.1.7), first detected in the UK in September 2020 (11). By December 2020, mutations N501Y, E484K, and K417N were reported, and classified as the Beta VOC (B.1.351). This variant was detected in South Africa (12), and it became the most dominant variant detected in 80% of SARS-CoV-2 genomes in the country. A month later, the Gamma variant (P.1) was reported in Brazil, as well as travelers from Brazil, arriving in Japan (13). In May 2021, a more infectious SARS-CoV-2 strain with increased mortality (14) spread rapidly through India and was termed the Delta variant (B.1.617.2). By December 2021, the Omicron variant was detected in South Africa, and rapidly spread around the world. From December 2021 to September 2023, the Omicron variant and its sub-lineages (BA.1, BA.2, BA.3, BA.4, BA.5, XBB, EG.5), including BA.1/BA.2 circulating recombinant forms (CRFs) are responsible for current COVID-19 cases worldwide (see footnote 2). Variant-defining mutations of these VoCs have functional implications with clinical significance which affect treatment and vaccine therapies. Thus, continuous characterization of SARS-CoV-2 in different populations is necessary since such data can be added to genomic repositories, and utilized to improve drug design and vaccine therapies.

One major method implemented in SARS-CoV-2 genetic characterization for detection of new circulating variants has been through genomic surveillance, which has mainly been achieved through the WGS of individual patient clinical samples. However, the drawback of this type of genomic surveillance is that data is only obtained from patients, who are tested in healthcare centers. Thus, SARS-CoV-2 genetic diversity in asymptomatic individuals, as well as those who do not seek attention in healthcare facilities, and some communities may be underestimated. Wastewater-based epidemiology (WBE) has proven to be an asset in the identification of COVID-19 hotspots and tracking the trends of infection in the community (15–17). Applying this population-based approach for SARS-CoV-2 genomic surveillance offers the added advantage of tracking the geographical distribution and predicting VoC occurrence in the population. Alongside WGS, allele specific genotyping (ASG) has been utilized as a tool for routine monitoring of SARS-CoV-2 variants in the population (18–20). Compared to whole genome sequencing, by next generation sequencing, allele-specific genotyping is less expensive and can be implemented on a larger scale in resource-limited settings. In this study, wastewater samples were used to describe the molecular epidemiology and genetic characteristics of SARS-CoV-2 in the Vhembe and Mopani districts, of Limpopo province, South Africa.

## 2 Materials and methods

### 2.1 Sample collection, processing and total RNA extraction

**Figure 1:**
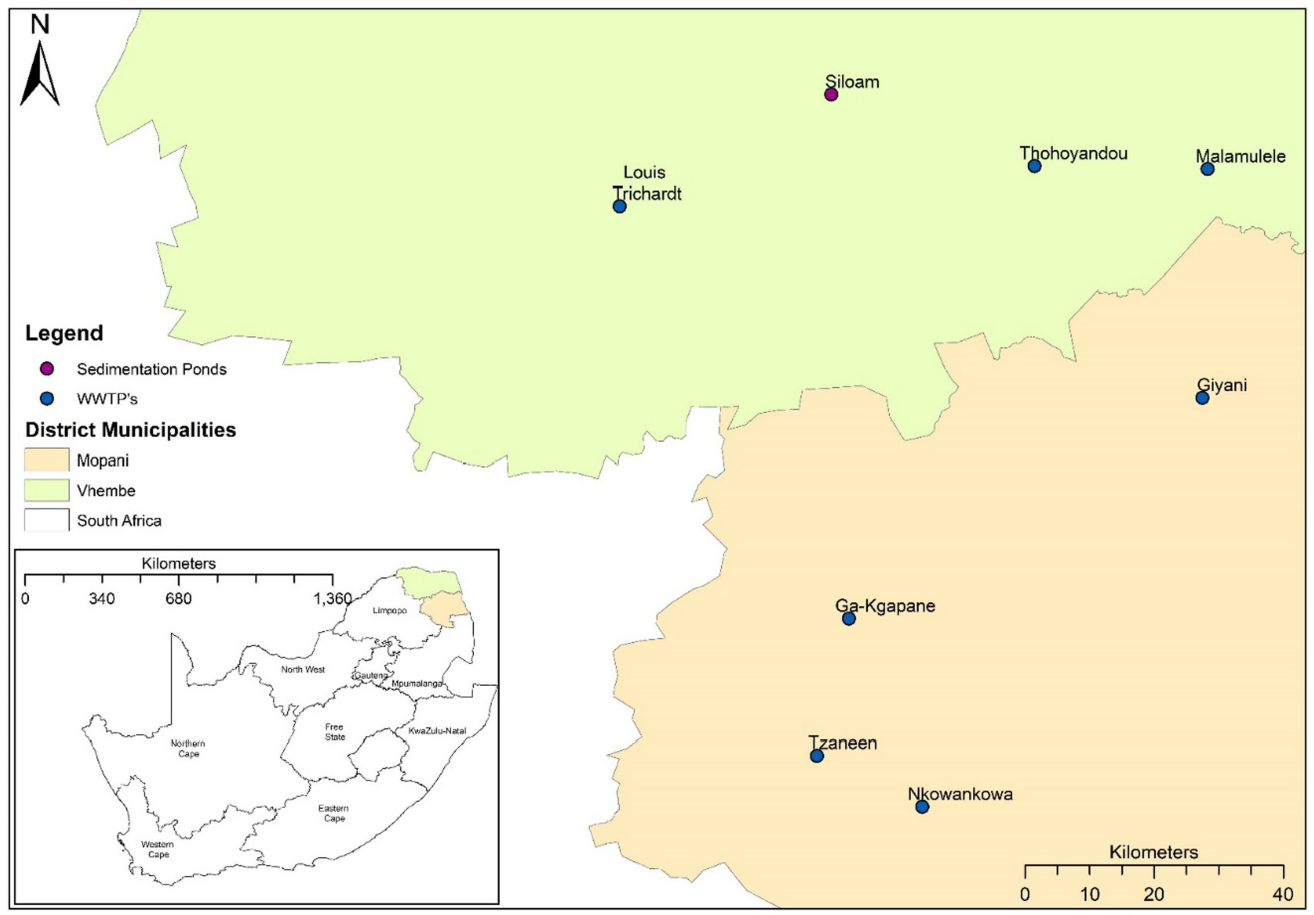
Map of South Africa indicating the wastewater treatment plants (WWTPs) and waste stabilization pond (WSP) in the Vhembe and Mopani districts.

Samples were collected from seven wastewater treatment plants (WWTPs) and one waste stabilization ponds (WSP) in the Vhembe and Mopani districts in Limpopo, South Africa (Figure 1). These WWTPs and WSPs were selected based on their functionality, accessibility and feasibility to collected repeated sampling based on resources available. Influent wastewater grab samples (500 mL) were collected at the raw inlet after the grid point from each of the sites once every week on a Monday over 17 months (January 2021 to May 2022). Samples were transported to the laboratory at 4°C and were processed for total RNA extraction. Samples were processed using a modified protocol described by Johnson et al. (21). Briefly, approximately 50–300 mL of wastewater influent (depending on the turbidity of the sample) was centrifuged at 3500 g for 20 min. The resulting pellet (~5 mL) was used for total RNA extraction using the QIAGEN RNeasy PowerSoil Kit (QIAGEN, Germany) according to the manufacturer’s protocol (‘RNeasy ® PowerSoil ® Total RNA Kit Handbook’, 2017). Total RNA concentration and purity were determined using a NanoDrop Spectrophotometer. The efficiency of this protocol has been described by (22).

### 2.2 SARS-CoV-2 quantification and variant of concern determination

#### 2.2.1 SARS-CoV-2 quantification by real-time PCR, quality control and results analysis

SARS-CoV-2 detection in wastewater samples was achieved by reverse transcription-quantitative polymerase chain reaction (RT-qPCR), using the iTaq Universal probes reaction mix one-step reaction kit (Bio-Rad Laboratories, Richmond, CA, USA) alongside primer/probe sets targeting the Nucleocapsid gene (N-gene). This was done using cycling conditions in a protocol developed by (23) and modified by (21). All reactions were performed in duplicates and run as a multiplex reaction in the QuantStudio™ 5 Real-Time PCR System. Analysis to determine the SARS-CoV-2 genome copy number in samples with positive amplification was done following a protocol previously described by (24).

#### 2.2.2 Allele-specific genotyping for SARS-CoV-2 mutation detection

To determine the circulating VoCs in the communities, genotypic analysis through an allele-specific qRT-PCR was performed for mutations pertaining to the Spike gene (S-gene) of SARS-CoV-2. For this study, SNP genotyping was done for some signatory mutations belonging to the Alpha, Beta, Delta, and Omicron VoCs. Only samples with SARS-CoV-2 concentration ≥ 1,500 g.c./mL were included for analysis, using the 7 TaqMan SARS-CoV-2 Mutation Panels, from ThermoFisher Scientific (Applied Biosystems), with the same cycling conditions as previously described by (19).

#### 2.2.3 Whole genome sequencing, genome assembly, lineage assignment and variant determination

SARS-CoV-2 RNA libraries were produced using the ATOPlex (MGI-Tech) protocol as previously described (19) and sequencing was done using the DNBSEQ-G400 instrument at the SAMRC Genomics Centre. Sequence data were analyzed using the Geneious version 2023.0 software as previously described (25). Consensus sequences were subjected to the Nextclade tool, for SARS-CoV-2 variant calling, clade assignment, and mutation determination for the viral genes. The Phylogenetic Assignment Named Global Outbreak LINeages (PangoLIN) interface is also in-built within the Nextclade database, for lineage assignment. Consensus sequences were also subjected to the COVID-19 Lineage Assigner PangoLIN tool for SARS-CoV-2 variant calling and lineage determination. SARS-CoV-2 variant calling and lineage assignment obtained from both tools were compared to confirm the assignment given. The phylogenetic relation between SARS-CoV-2 genomes from this study and the retrieved full-length SARS-CoV-2 genomes was determined by phylogenetic analysis, using the MEGA 11 software (neighbor-joining method). The proportion of duplicates was calculated using 1,000 bootstraps replicate.

#### 2.2.4 Genetic diversity of SARS-CoV-2 viruses in the study sites compared to those around the world

Previously published full-length SARS-CoV-2 sequences from the Limpopo province, South Africa, and other countries classified as Alpha, Beta, Delta, and Omicron VoCs were downloaded from the Global Initiative on Sharing Avian Influenza Data (GISAID) database. These previously published SARS-CoV-2 sequences (henceforth referred to as “reference sequences”), were imported to the Geneious v2023.0 software, and aligned with study sequences having similar VoC assignment, using the MAFFT v7.490 parameters. Genetic diversity of each variant was determined by comparing the mutations present in the study sequence to those in the reference sequence. This was done for all four VoCs detected in the study site. Furthermore, the MEGA 11 software was used to compute the estimates of evolutionary divergence between sequences.

#### 2.2.5 Comparison between allele-specific variant genotyping and WGS in VoC determination

Samples that were subjected to allele-specific variant genotyping and WGS were compared to infer whether they yielded similar VoC assignments. Key mutations in the Spike gene coding for Alpha (N501Y, DelH69V70, P681H), Beta (N501Y, E484K, K417N), Delta (L452R, P681R) and Omicron (N501Y, DelH69V70, P681H, K417N) VoCs, were used for SNP VoC determination. For samples subjected to WGS, VoC assignment was determined by Nextclade. To determine whether samples subjected to both techniques had the same variant call, the presence of key mutations in the S-gene (using the allele-specific genotyping criteria) were investigated for both techniques.

## 3 Results

### 3.1 Molecular epidemiology of SARS-CoV-2 in the Vhembe and Mopani districts (January 2021 to May2022)

Out of 487 samples collected from eight wastewater treatment sites, 75% (365/487) were positive for SARS-CoV-2 RNA by qRT-PCR. Of these, 80 met ASG criteria. One-fifth (75/365) of the SARS-CoV-2 positive samples detected throughout the 17 months’ surveillance period (January 2021 to May 2022) were used for WGS. Eighty percent (60/75) of these sequences passed QC and were successfully analyzed using the Nextclade and PangoLIN software. These sequences are submitted to the NCBI SARS-CoV-2 SRA database, under project number: PRJNA980445.^3^

Full length genome sequences obtained from the ATOPlex MGI sequencing platform, showed that the Delta variant was most dominant (45%) across the study sites, closely followed by the Omicron variant (31.7%) throughout the surveillance period. The Beta VoC occurred at low frequencies (5%), while the Alpha VoC was not detected in any of the study sites. Both tools (PangoLIN and Nextclade) did not assign a specific VoC name for 18% (11/60) of the study sequences, but assigned the lineage and clade for these sequences, and thus were designated as “unassigned,” for the purpose of classification in this study.

**Figure 2:**
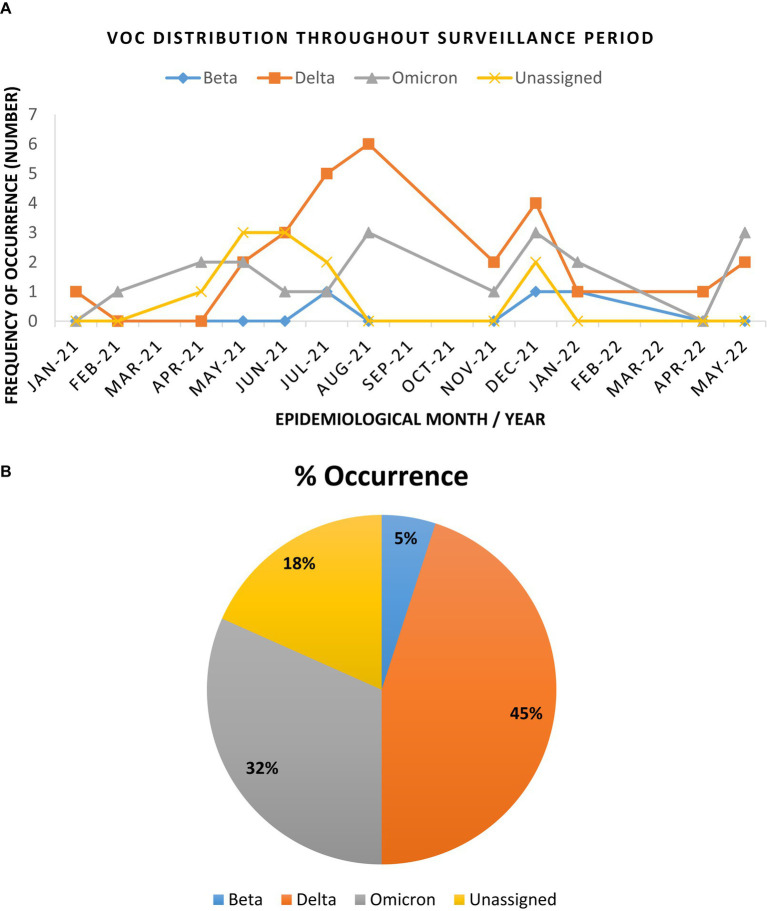
Trend and distribution of full genome SARS-CoV-2 variants of concern in the Vhembe and Mopani districts during the 17 months’ study period. **(A)** Distribution of SARS-CoV-2 VoCs between January 2021 to May 2022. The Delta variant was most dominant between April and August 2021, followed by Omicron which was more prominent between December 2021 and January 2022; the “unassigned” variants were most prominent between April and July 2021, while the Beta variants were sparsely detected between July and December 2021. **(B)** Pie chart illustrating the cumulative frequency of variant occurrence.

The Beta VoC was only sparsely observed between July – December 2021, as well as in January 2022. Interestingly, Delta and Omicron VoCs were detected during this phasing out of the second wave. This was observed in January and February 2021 for the Delta and Omicron VoCs, respectively (Figure 2). As surveillance continued, the Delta VoC circulation was dominant in the study sites and was most prevalent between April – August 2021. Omicron VoC was also in continuous circulation at all sites but only became more prominent between December 2021 and January 2022. Figure 2 illustrates the distribution of the VoCs observed throughout the surveillance period and the overall occurrence of the detected variants.

### 3.2 Genetic characteristics of SARS-CoV-2 in the study sites

The obtained SARS-CoV-2 whole genomes sequences ranged between 29,842–29,903 kilobases (kb) for the obtained 60 viruses throughout the surveillance. The identified Beta, Delta, Omicron, and “unassigned” variants belonged to 12 lineages and 11 clades. The lineages detected include: B.1, B.1.1, B.1.1.174, B.1.351, B.1.617, B.1.617.2, B.1.1.529, AY.45, BA.1, BA.2, BA.4, BE.1 (alias BA.5.3.1.1). Lineage AY.45, associated with the Delta variant was first detected in January 2021, while lineage B.1.1.529 associated with the Omicron variant first occurred in February 2021 (see Table 1).

**Table 1:** Frequency of occurrence of Lineages occurring in the study sites throughout the study period.

Lineages	Frequency of occurrence
Delta variant (AY.45 and B.1.617.2)	45%
AY.45 Lineage (30%)	
B.1.617.2 Lineage (15%)	
Omicron Variant (B.1.1.529, BA.1, BA.2, BA.4, and BE.1)	32%
BA.1 lineage (47.4%)	
BA.2 lineage (10.5%)	
BA.3 (also known as B.1.1.529) lineage (15.7%)	
BA.4 lineage (21%)	
BA.5 (5.3%)	
Beta Variant (B.1.351)	5%
“Unassigned” Variant (B.1, B.1.1, B.1.617, and B.1.1.174)	18%

**Figure 3:**
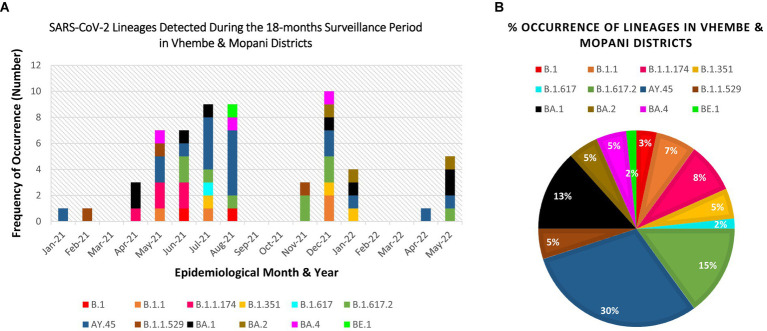
Distribution and percentage occurrence of SARS-CoV-2 lineages detected at the study sites. Lineage B.1.351 represents the Beta VoC; B.1.617.2, AY.39, AY.45 represent the Delta VoC; B.1.1.529, BA.1, BA.2, BA.4, BE.1 represent the Omicron VoC. The remaining lineages B.1, B.1.1, B.1.617, and B.1.1.174 represent the “unassigned” variants. **(A)** Illustration of the diversity of lineages detected at different time points of assessment. **(B)** Overall percentage occurrence of each of the 12 lineages detected throughout the surveillance period. (NB: Sequences were not available for Mar-21, Sep-21, Oct-21, Feb-22, Mar-22).

**Figure 4:**
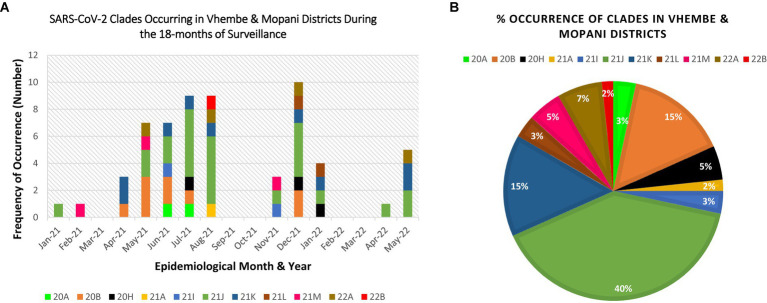
Distribution and percentage occurrence of SARS-CoV-2 clades detected at the study sites. Distribution and percentage occurrence of SARS-CoV-2 lineages detected in the study sites. Clade 20H represents the Beta VoC; 21A, 21I, 21J represent the Delta VoC; 21K, 21L, 21M, 22A, 22B represent the Omicron VoC. The remaining clades (20A and 20B) represent the “unassigned” variants. Fig **(A)** illustrates the diversity of lineages detected at different time points of assessment. Fig **(B)** highlights the overall percentage occurrence of each of the 11 clades detected throughout the surveillance period. (NB: Sequences were not available for Mar-21, Sep-21, Oct-21, Feb-22, Mar-22).

Eleven clades namely: 20A, 20B, 20H, 21A, 21I, 21J, 21K, 21L, 21M, 22A, and 22B were detected at the study sites throughout the surveillance period. Clade 21J, associated with the Delta variant was first observed in January 2021, while Clade 21 M associated with the Omicron variant first occurred in February 2021 (see Table 2). Figures 3, 4 illustrate the distribution and frequency of the lineages and clades detected.

**Table 2:** Frequency of occurrence of clades occurring in the study sites throughout the study period.

Clades	Frequency of occurrence
Delta Variant (21A, 21I, and 21 J)	45%
Clade 21A (1.7%)	
Clade 21I (3.3%)	
Clade 21J (40%)	
Omicron Variant (21K, 21L, 21M, 22A, and 22B)	32%
Clade 21K (15%)	
Clade 21L (3.3%)	
Clade 21M (5%)	
Clade 22A (6.7%)	
Clade 22B (1.7%)	
Beta variant (20H)	5%
“Unassigned” Variant (20A and 20B)	18%

**Figure 5:**
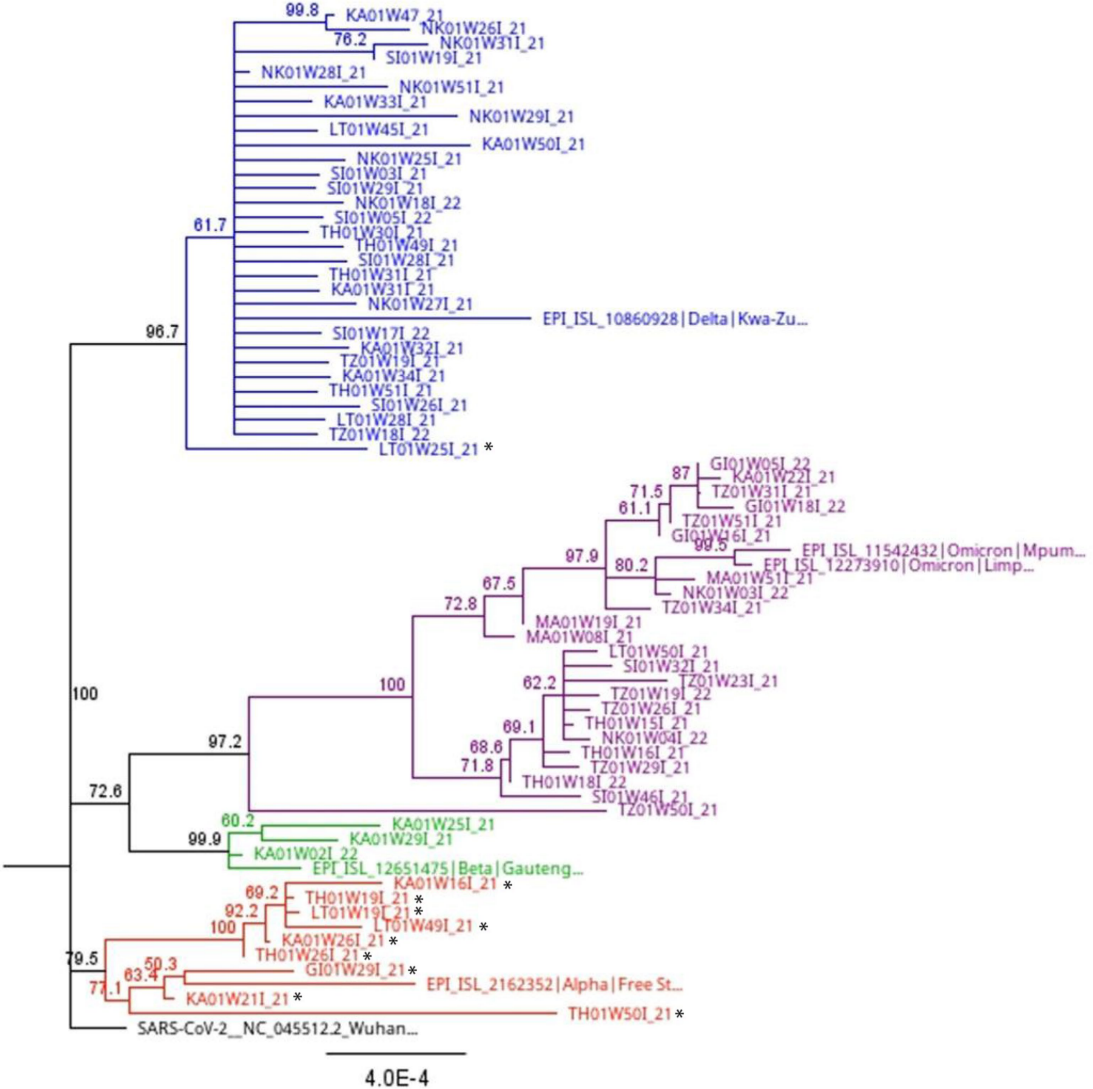
Full length phylogenetic relationship between study sequences and reference sequences (indicated with colored shapes) from South Africa with 1,000 bootstrap iterations. The blue branches highlight all lineages (B.1.617.2, AY.39, AY.45) and clades (21A, 21I, 21J) associated with the Delta variant. The purple branches show all lineages (B.1.1.529, BA.1, BA.2, BA.4, BE.1) and clades (21K, 21L, 21M, 22A, 22B) associated with the Omicron variant. The green branches highlight lineage B.1.351 and clade 20H associated with the Beta variant. The red branches highlight lineage B.1.1.7 and clade 20I associated with the Alpha variant. Sequences with a black star are those assigned a lineage (B.1, B.1.1, B.1.617, and B.1.1.74) and clade (20A and 20B) by the Nextclade and PangoLIN tools, but not a specific variant name. Phylogenetic analysis shows some of these sequences clustering with the Alpha and Delta variants. However, others “unassigned” sequences still clustered with each other.

Phylogenetic analysis of full-length sequences was applied to corroborate the results obtained through variant, lineage and clade assignment obtained from the PangoLIN and Nextclade tools, as well as determine the closest relationship of the 11 sequences that were “unassigned” using the whole genome sequencing method. Interestingly, these “unassigned” study sequences clustered with Alpha and Delta variant sequences. Specifically, 2/11 (18.2%) “unassigned” study sequences clustered with Delta variant study and reference sequences. Three “unassigned” study sequences (3/11; 27.3%) clustered with Alpha variant reference sequences, while the remaining 7/11 (54.5%) “unassigned” study sequences clustered with each other (Figure 5).

### 3.3 Full length intra-variant genetic diversity among study sequences

Investigation of the intra-variant genetic diversity among the study sequences belonging to the same variant showed little variability occurring within them. Among the Beta variant sequences, the intra-genetic variability ranged between 0.0003 and 0.0018. Similarly, minor differences in genetic diversity was observed between the Delta (0.00–0.0012) and Omicron (0.00–0.0018) variant study sequences. Among the unassigned study sequences, however, a slightly higher variability (0.00–0.0022) was observed.

### 3.4 Mutations in the S-protein receptor-binding domain

A total of 12 mutations were detected in the receptor-binding domain (RBD) of the Beta variant study sequences, with two of them (K417N and N501Y) occurring at a higher frequency. Among the Delta variant study sequences, two previously described RBD mutations (L452R and T478K) occurred at a higher frequency compared to the three novel mutations (A361S, V327I, D427Y) also detected in some sequences (Table 3). Within the RBD of the Omicron study sequences, 18 common mutations were detected, the highest among all the variants. However, mutations D405N and R408S, which are commonly detected in lineages BA.2, BA.4, and BA.5 were completely absent in the study sequences classified as BA.2, BA.4, and BA.5 lineages. Of the 11 unassigned study sequences, 2/11 (18.2%) had no mutations in the RBD region, whereas, in the other 9/11 (81.8%), mutation Q498H was the most prevalent. Details of the frequency of occurrence of mutations detected in the RBD are presented in Table 3.

**Table 3:** Frequency of occurrence of S-protein RBD mutations in the study sequences.

Beta S-protein RBD*		Delta S-protein RBD*		Omicron S-protein RBD*		Unassigned S-protein RBD*	
Mutation	Frequency	Mutation	Frequency	Mutation	Frequency	Mutation	Frequency
G339D	1/3 (33.3%)	A361S	2/27 (7.4%)	G339D	19/19 (100%)	A372T	1/11 (9%)
K417N	3/3 (100%)	V327I	1/27 (3.7%)	S371L	11/19 (57.8%)	K417T	2/11 (18.2%)
N440K	1/3 (33.3%)	D427Y	1/27 (3.7%)	S371F	5/19 (26.3%)	S477N	1/11 (9%)
K444-	1/3 (33.3%)	L452R	26/27 (96.3%)	S373P	18/19 (94.7%)	T478K	2/11 (18.2%)
V445-	1/3 (33.3%)	L452W	1/27 (3.7%)	S375F	17/19 (89.4%)	E484Q	1/11 (9%)
S477N	1/3 (33.3%)	T478K	27/27 (100%)	T376A	6/19 (31.5%)	Q493K	1/11 (9%)
T478K	1/3 (33.3%)	Q498H	1/27 (3.7%)	K417N	18/19 (94.7%)	Q498H	6/11 (54.5%)
E484A	1/3 (33.3%)			N440K	11/19 (57.8%)		
Q493R	1/3 (33.3%)			G466S	8/19 (42.1%)		
Q498K	1/3 (33.3%)			L452R	5/19 (26.3%)		
P499S	1/3 (33.3%)			S477N	8/19 (42.1%)		
N501Y	3/3 (100%)			T478K	19/19 (100%)		
				E484A	12/19 (63.2%)		
				F486V	5/19 (26.3%)		
				Q493R	13/19 (68.4%)		
				Q498R	18/19 (94.7%)		
				N501Y	18/19 (94.7%)		
				Y505H	18/19 (94.7%)		

*RBD, receptor-binding domain.

### 3.5 Genetic diversity within the S-protein RBD

Beta variant study sequences (*n* = 3) were compared to previously published Beta variant sequences obtained from GISAID. These reference sequences originated from the Limpopo province (*n* = 4), South Africa (*n* = 9), other African nations (*n* = 29), the Americas (*n* = 2), Europe (*n* = 15), Asia, and the Middle East (*n* = 22). Mutation E484K, has been associated with reduced neutralizing activity of human polyclonal sera induced in convalescent and vaccinated individuals (26). This mutation was absent in all Beta variant study sequences, but was present in all reference sequences (see Table 4). The average evolutionary divergence between the Beta variant study and reference sequences was estimated to be 0.0006, showing similarity between them.

**Table 4:** Frequency of occurrence of key mutations at the RBD of the S-protein defining the Beta VoC between different viral populations from different countries or continent.

SARS-CoV-2 VOC	S-gene defining mutations	Vhembe and Mopani District (%)	Limpopo (%)	South Africa (%)	African Nations (%)	Americas (%)	Europe (%)	Asia and Middle East (%)
Beta variant	K417N	100	100	100	93.1	100	100	100
	E484K	0	100	100	96.6	100	100	100
	N501Y	100	100	88.9	96.6	100	93.3	90.9
Delta variant	L452R	96.2	100	100	100	100	100	100
	L452W	3.7	0	0	0	0	0	0
	T478K	96.2	100	100	100	100	100	100
	V327I	3.7	0	0	0	0	0	0
	A361S	7.4	0	0	0	0	0	0
	D427Y	3.7	0	0	0	0	0	0
Alpha variant	N501Y	0	100	100	97.5	100	100	100

Delta variant study sequences (27) were compared to reference Delta variant sequences (*n* = 71) from GISAID. These previously published sequences originated from the Limpopo province (*n* = 7), South Africa (*n* = 12), other African nations (*n* = 32), the Americas (*n* = 4), Europe (*n* = 12), Asia, and the Middle East (*n* = 9). One out of 27 (3.7%) of the Delta variant study sequences, carried the amino acid (aa) Tryptophan (W) in place of Arginine (R) at position 452. Three previously undescribed novel mutations (V327I, A361S, and D427Y) were detected in the study sequences, but not the reference sequences. The evolutionary divergence between the study and reference sequences was estimated to be 0.0008, showing a close similarity between the sequences (see Table 4).

Omicron study sequences were compared to 54 Omicron reference sequences obtained from GISAID. The proportion of Omicron lineages downloaded was as follows: 7/54 (12.9%) were of BA.1 lineage, 25/54 (46.3%) for BA.2, 2/54 (3.7%) sequences were of BA.4 lineage and BA.5 occurred at 20/54 (37%). Of the 18 RBD mutations in the Omicron variant, only mutations D405N and R408S, belonging to the BA.2, BA.4, and BA.5 lineages, were completely absent in the study sequences. These mutations are known to evade humoral immunity elicited by Omicron BA.1 infection. However, they were present at high frequencies in the reference sequences. Even with these differences, the average evolutionary divergence (0.0015) between the Omicron study and reference sequences was low.

“Unassigned” study sequences which clustered with the Alpha variant (*n* = 9) after phylogenetic analysis (Figure 5) were compared to Alpha variant reference sequences originating from Limpopo province (*n* = 4), South Africa (*n* = 8), other African nations (*n* = 40), the Americas (*n* = 4), Europe (*n* = 13), Asia, and the Middle East (*n* = 13). Mutation N501Y was the only common mutation found in the RBD of the study and reference sequences. This mutation increases ACE2 binding affinity, causing the virus to become more infectious. This mutation was completely absent in the study sequences, but present at high frequency (>60%) in the other populations. The average evolutionary divergence (0.001) between the study and reference sequences was also low.

### 3.6 Allele-specific variant genotyping versus WGS in VoC determination

Of the 80 samples that met the criteria for allelic variant genotyping, 41/80 (51.3%) were subjected for whole genome sequencing. For 21/41 (51.2%) samples evaluated by both techniques, concordance was observed between the S-gene-defining mutations and variant assignment. For 13/41 (31.7%) samples, at least one S-gene defining mutation was observed in both techniques, but with a different variant assignment. Interestingly, there were 7/41 (17%) samples in which no concordance existed between mutations detected by allelic variant genotyping or variant assignment in both techniques.

## 4 Discussion

Wastewater-based genomic surveillance of SARS-CoV-2 provides a comprehensive approach to characterize evolutionary patterns and distribution of viral types in a population, since wastewater is known to contain an aggregate of SARS-CoV-2 viruses from multiple individuals, which occur at low concentrations in various states of genomic integrity. In this study, wastewater samples were used to describe the molecular epidemiology and genetic characteristics of SARS-CoV-2 in two districts (Vhembe and Mopani), of South Africa. The Delta and the Omicron VoCs were detected in the study sites by January and February of 2021, respectively, predating reports from the South African National Institute of Communicable Diseases (NICD) which documented the appearance of these variants in the country in May and November 2021, respectively (27). Both variants were detected in the study sites toward the end of the second wave (January – February 2021) when the Beta variant was still predominant in South Africa.

Nine lineages and nine clades were identified at the study sites throughout the surveillance period. Lineage AY.45 or B.1.617.2 (Clade 21J) was the most dominant lineage and mostly predominated during the third wave (May – September 2021) of infections in South Africa, the South African National Institute for Communicable Disease (NICD) reported (See footnote 4). The fourth wave in South Africa which began on 06 December 2021 saw the predominance of the Omicron VoC among the population, with lineage BA.1 being responsible for most infections in the population. Earlier reports of the BA.1 lineage occurrence in the population indicate that this lineage spread from the Gauteng province to other provinces in South Africa, and to two regions of Botswana from late October to November 2021 (28). Interestingly, our data shows that this variant was circulating in the study population as early as April 2021 (Supplementary Table S1), and its dominance (47.4%) occurred throughout the surveillance period. Our findings are contrary to other wastewater-based surveillance studies conducted in Cape Town, South Africa, which reported the complete replacement of lineage BA.1 with lineage BA.2 by mid-January 2022 in 31 WWTPs (19). The first appearance of lineage BA.4 likely occurred in mid-December 2021, with phylogeographic analysis indicating probable dispersal from Limpopo province to Gauteng province, and subsequently to other provinces. Similarly, lineage BA.5 is reported to have emerged in early January 2022, and dispersed from the Gauteng province to other provinces in South Africa (29). In our study, the earliest detection of lineage BA.4 was in May 2021, while lineage BA.5 was observed by August 2021. These observations highlight the advantage of using WBE as a surveillance approach for early detection of lineages that were already circulating in the population, but only became dominant in individuals much later. In addition, the little intra-variant genetic diversity between the study sequences and previously published reference sequences further corroborates the silent circulation of these lineages prior to detection in individuals. Similar observations of early detection of cryptic lineages through wastewater surveillance studies have also been previously reported (30, 31), where nonsynonymous mutations detected in wastewater only became dominant in the population at a later stage of the COVID-19 epidemic.

In terms of genetic diversity within the Spike gene RBD of the study sequences, some peculiarities were observed. For example, mutation E484K in the Beta variant was absent from all the Beta variant study sequences. Mutation E484K in the RBD of the Beta variant enhances viral binding affinity to human ACE2, as well as reduced antibody neutralizing effect in convalescent and vaccinated individuals (26). This is relevant because the S-protein RBD facilitates SARS-CoV-2 infectivity, transmission, and antibody-mediated neutralization (32–35). Thus, the absence of this mutation in our Beta variant study sequences may explain why the Beta variant was sparsely detected (5%) in our study sites. Secondly, three novel mutations were detected in the RBD of the Delta variant. Investigating the implication of these mutations is needed to understand their role in viral infectivity and pathogenicity. Next, mutations L425R and T478K in the RBD of the Delta variant are associated with increased affinity with ACE2 (36). While these mutations occurred at high frequencies (96.3%) in Delta variant study sequences, a change in amino acid at position 452 (R→W; L452W) was also observed, though at a lower frequency (3.7%). This new change, alongside the three previously undescribed mutations (V327I, A361S, and D427Y) require further investigation. Reports have shown that, while some neutralizing antibodies are effective against BA.2.12.1, BA.4 and BA.5 Omicron subvariants, mutations S371F, D405N and R408S undermine most sarbecovirus-neutralizing antibodies (37). The absence of mutations D405N and R408S in the RBD from all the Omicron sequences from the current study have several implications. First, while this study showed occurrence of the Omicron variant in the study sites as early as February 2021, the absence of these mutations may have probably influenced its continuous, but dormant circulation in the population. Secondly, the absence of these mutations may explain why the fourth wave of COVID-19 infections, characterized by the Omicron VoC had a decreased severity in the study area. Although high SARS-CoV-2 viral loads were detected in wastewater in the study sites, fewer clinical cases were reported. This may have been due to an increase in vaccine uptake in these communities.

The S-gene RBD of study sequences which the PangoLIN and Nextclade tools only assigned lineages and clades revealed the absence of specific variant defining-mutations which are used in classifying SARS-CoV-2 strains belonging to a specific variant. This may have been the reason why they were only assigned lineages and clades, but not a specific variant name. Mutation Q498H was the most common mutation of these “unassigned variants.” The presence of this mutation is associated with increased binding affinity of the viral spike protein to the ACE2 receptor, which facilitates viral entry during (38). The presence of this mutation also boosts binding of other RBD variants, which could imply an increased infectivity for the population in the presence of this mutation.

Utilizing the current data to further investigate minority variants occurring at lower thresholds in the Spike RBD could potentially predict the next nonsynonymous mutations that may generate another lineage, which may occur in the population. This is relevant because, although the WHO has announced the end of the COVID-19 pandemic, new Omicron subvariants are constantly emerging, with the latest being of lineage (39), as of July 2023. This highlights the need for constant genomic surveillance, at a population level. Additionally, it could also contribute to vaccine development efforts (40), as well as facilitate designation of improved ASG panels. In South Africa, population-based genomic surveillance through WBE is led by the South African Collaborative COVID-19 Surveillance System (SACCESS) network, which was established in 2021. It operates in collaboration with the NICD and the South African Medical Research Council (SAMRC). The goal of this network is to develop standard methodology for the identification and sequencing of SARS-CoV-2 from wastewater (41). This nationwide wastewater surveillance is comparable to what has been established in other nations such as the Netherlands, Australia, England, Turkey (42), and the European 100 cities program. These systems have been implemented by the governmental public health arms of these nations for monitoring SARS-CoV-2 occurrence which will serve as an early warning system, and aid with public health policy decisions.

Allele-specific genotyping has been shown to be a cost-effective method for monitoring variants (43). Our findings indicate that variant assignment determined by allele-specific or single nucleotide polymorphism (SNP) genotyping was 51.2% accurate when compared to results obtained through WGS. This low accuracy could be due to the fact that the presence of at least one mutation does not necessarily prove the occurrence of a variant, since these variants share ≥1 mutation (44). In this study, mutations pertaining to the S-gene were used to detect the occurrence of Alpha, Beta, Delta, and Omicron VoCs in the study sites. The N501Y mutation is shared by all variants except Delta; delH69V70 and mutation P681H are common to both Alpha and Omicron variants; K417N is common to both Beta and Omicron, while mutation L452R is present in both the Delta variant and Omicron BA.4 and BA.5 lineages. This could lead to assigning more than one variant per sample, which may not be a true reflection of variant occurrence. To optimize this technique, and improve variant calling, mutations specific to each variant could be included (45).

In conclusion, the current study demonstrates that population-based approaches in genomic surveillance may be advantageous over individual-specific approaches. This study has shown that Delta and Omicron lineages were in circulation in the population earlier than previous reports from South Africa have stated. Furthermore, genetic characterization of SARS-CoV-2 in the study sites has revealed novel mutations whose implications need further investigation.

## Data availability statement

The datasets presented in this study can be found in online repositories. The names of the repository/repositories and accession number(s) can be found in the article/Supplementary material.

## Ethics statement

The study protocol was approved by the Animal, Environmental and Biosafety Research Ethics Committee at the University of Venda (SMNS/20/MBY/14/0903).

## Author contributions

LT: Data curation, Formal analysis, Investigation, Methodology, Writing – original draft. PM: Investigation, Methodology, Writing – review & editing. NM: Data curation, Formal analysis, Methodology, Writing – review & editing. MM: Investigation, Methodology, Writing – review & editing. JE: Data curation, Investigation, Methodology, Writing – review & editing. AV: Investigation, Methodology, Writing – review & editing. BG: Methodology, Writing – review & editing, Investigation. DT: Data curation, Investigation, Methodology, Writing – review & editing. LM-R: Data curation, Investigation, Supervision, Writing – review & editing. RS: Conceptualization, Funding acquisition, Resources, Writing – review & editing. RJ: Conceptualization, Funding acquisition, Formal analysis, Resources, Writing – review & editing. CK: Conceptualization, Funding acquisition, Formal analysis, Resources, Writing – review & editing. PB: Conceptualization, Funding acquisition, Resources, Writing – review & editing.

## Footnotes

^3^https://www.ncbi.nlm.gov/sra/PRJNA980445

## References

[1] COVID-Cornavirus Statistics Worldometer (2023) Available at: https://www.worldometers.info/coronavirus/

[2] Ahmad SU Hafeez Kiani B Abrar M Jan Z Zafar I Ali Y. A comprehensive genomic study, mutation screening, phylogenetic and statistical analysis of SARS-CoV-2 and its variant omicron among different countries. J Infect Public Health. (2022) 15:878–91. doi: 10.1016/j.jiph.2022.07.002, PMID: 35839568 PMC9262654

[3] O’Toole Á Scher E Underwood A Jackson B Hill V McCrone JT. Assignment of epidemiological lineages in an emerging pandemic using the pangolin tool. Virus Evol. (2021) 7:1–9. doi: 10.1093/ve/veab064, PMID: 34527285 PMC8344591

[4] Rambaut A Holmes EC O’Toole Á Hill V McCrone JT Ruis C. A dynamic nomenclature proposal for SARS-CoV-2 lineages to assist genomic epidemiology. Nat Microbiol. (2020) 5:1403–7. doi: 10.1038/s41564-020-0770-5, PMID: 32669681 PMC7610519

[5] Abavisani M Rahimian K Mahdavi B Tokhanbigli S Mollapour Siasakht M Farhadi A. Mutations in SARS-CoV-2 structural proteins: a global analysis. Virol J. (2022) 19:220–19. doi: 10.1186/s12985-022-01951-7, PMID: 36528612 PMC9759450

[6] Grubaugh ND Petrone ME Holmes EC. We shouldn’t worry when a virus mutates during disease outbreaks. Nat Microbiol. (2020) 5:529–30. doi: 10.1038/s41564-020-0690-4, PMID: 32071422 PMC7095397

[7] Lauring AS. Genetic variants of SARS-CoV-2 — what do they mean? JAMA. (2021) 325:529–31. doi: 10.1001/jama.2020.27124, PMID: 33404586

[8] V’kovski P Kratzel A Steiner S Stalder H Thiel V. Coronavirus biology and replication: implications for SARS-CoV-2. Nat Rev Microbiol. (2020) 19:155–70. doi: 10.1038/s41579-020-00468-6, PMID: 33116300 PMC7592455

[9] World Health Organization (2023) Available at: https://www.who.int/activities/tracking-SARS-CoV-2-variants

[10] Korber B Fischer WM Gnanakaran S Yoon H Theiler J Abfalterer W. Article tracking changes in SARS-CoV-2 spike: evidence that D614G increases infectivity of the COVID- ll tracking changes in SARS-CoV-2 spike: evidence that D614G increases infectivity of the COVID-19 virus (2020) 182:812–827.e19. doi: 10.1016/j.cell.2020.06.043, PMID: 32697968 PMC7332439

[11] Meng B Kemp SA Papa G Datir R Ferreira IATM Marelli S. Recurrent emergence of SARS-CoV-2 spike deletion H69/V70 and its role in the alpha variant B.1.1.7. Cell Rep. (2021) 35:109292. doi: 10.1016/j.celrep.2021.109292, PMID: 34166617 PMC8185188

[12] Tegally H Wilkinson E Giovanetti M Iranzadeh A Fonseca V Giandhari J. Detection of a SARS-CoV-2 variant of concern in South Africa. Nature. (2021) 592:438–43. doi: 10.1038/s41586-021-03402-9 33690265

[13] Ramundo M. S.. (2021) Genomics and epidemiology of the P.1 SARS-CoV-2 lineage in Manaus, Brazil, 821, 815–821. 10.1126/science.abh2644 PMC8139423 33853970

[14] Cherian S Potdar V Jadhav S Yadav P Gupta N das M. Sars-cov-2 spike mutations, l452r, t478k, e484q and p681r, in the second wave of covid-19 in Maharashtra, India. Microorganisms. (2021) 9:1–11. doi: 10.3390/microorganisms9071542, PMID: 34361977 PMC8307577

[15] Ahmed W Angel N Edson J Bibby K Bivins A O'Brien JW. First confirmed detection of SARS-CoV-2 in untreated wastewater in Australia: a proof of concept for the wastewater surveillance of COVID-19 in the community. Sci Total Environ. (2020) 728:138764. doi: 10.1016/j.scitotenv.2020.138764, PMID: 32387778 PMC7165106

[16] Randazzo W Truchado P Cuevas-Ferrando E Simón P Allende A Sánchez G. SARS-CoV-2 RNA in wastewater anticipated COVID-19 occurrence in a low prevalence area. Water Res. (2020) 181:115942. doi: 10.1016/j.watres.2020.115942, PMID: 32425251 PMC7229723

[17] la Rosa G Iaconelli M Mancini P Bonanno Ferraro G Veneri C Bonadonna L. First detection of SARS-CoV-2 in untreated wastewaters in Italy. Sci Total Environ. (2020) 736:139652. doi: 10.1016/j.scitotenv.2020.139652, PMID: 32464333 PMC7245320

[18] Harper H Burridge A Winfield M Finn A Davidson A Matthews D. Detecting SARS-CoV-2 variants with SNP genotyping. PLoS One. (2021) 16:1–12. doi: 10.1371/journal.pone.0243185, PMID: 33626040 PMC7904205

[19] Johnson R Mangwana N Sharma JR Muller CJF Malemela K Mashau F. Delineating the spread and prevalence of SARS-CoV-2 omicron sublineages (BA.1-BA.5) and Deltacron using wastewater in the Western cape, South Africa. J Infect Dis. (2022) 226:1418–27. doi: 10.1093/infdis/jiac356, PMID: 36017801 PMC9574669

[20] Takemae N Doan YH Momose F Saito T Kageyama T. Development of new SNP genotyping assays to discriminate the omicron variant of SARS-CoV-2. Jpn J Infect Dis. (2022) 75:411–4. doi: 10.7883/yoken.jjid.2022.007, PMID: 35095027

[21] Johnson R Muller CJF Ghoor S Louw J Archer E Surujlal-Naicker S. Qualitative and quantitative detection of SARS-CoV-2 RNA in untreated wastewater in Western Cape Province, South Africa. S Afr Med J. (2021) 111:198–202. doi: 10.7196/SAMJ.2021.V111I3.15154, PMID: 33944737

[22] Johnson R Sharma JR Ramharack P Mangwana N Kinnear C Viraragavan A. Tracking the circulating SARS-CoV-2 variant of concern in South Africa using wastewater-based epidemiology. Sci Rep. (2022) 12:1182. doi: 10.1038/s41598-022-05110-4, PMID: 35064174 PMC8783013

[23] Peccia J Zulli A Brackney DE Grubaugh ND Kaplan EH Casanovas-Massana A. Measurement of SARS-CoV-2 RNA in wastewater tracks community infection dynamics. Nat Biotechnol. (2020) 38:1164–7. doi: 10.1038/s41587-020-0684-z, PMID: 32948856 PMC8325066

[24] Street R Mathee A Mangwana N Dias S Sharma JR Ramharack P. Spatial and temporal trends of SARS-CoV-2 RNA from wastewater treatment plants over 6 weeks in Cape Town, South Africa. Int J Environ Res Public Health. (2021) 18:1–9., PMID: 34831841 10.3390/ijerph182212085 PMC8618134

[25] Matume ND Tebit DM Gray LR Hammarskjold ML Rekosh D Bessong PO. Next generation sequencing reveals a high frequency of CXCR4 utilizing viruses in HIV-1 chronically infected drug experienced individuals in South Africa. J Clin Virol. (2018) 103:81–7. doi: 10.1016/j.jcv.2018.02.008, PMID: 29661652 PMC7229640

[26] Jangra S Ye C Rathnasinghe R Stadlbauer D Krammer F Simon V. SARS-CoV-2 spike E484K mutation reduces antibody neutralisation. Lancet Microbe. (2021) 2:e283–4. doi: 10.1016/S2666-5247(21)00068-9, PMID: 33846703 PMC8026167

[27] NICD (2022) SARS-CoV-2 genomic surveillance update - NICD’, (July). Available at: https://www.nicd.ac.za/diseases-a-z-index/disease-index-covid-19/sars-cov-2-genomic-surveillance-update/. (accessed 10 November 2022)

[28] Viana R Moyo S Amoako DG Tegally H Scheepers C Althaus CL. Rapid epidemic expansion of the SARS-CoV-2 omicron variant in southern Africa. Nature. (2022) 603:679–86. doi: 10.1038/s41586-022-04411-y, PMID: 35042229 PMC8942855

[29] Tegally H Moir M Everatt J Giovanetti M Scheepers C Wilkinson E. Emergence of SARS-CoV-2 omicron lineages BA.4 and BA.5 in South Africa. Nat Med. (2022) 28:1785–90. doi: 10.1038/s41591-022-01911-2, PMID: 35760080 PMC9499863

[30] Gregory DA Trujillo M Rushford C Flury A Kannoly S San KM. Genetic diversity and evolutionary convergence of cryptic SARS- CoV-2 lineages detected via wastewater sequencing. PLoS Pathog. (2022) 18:e1010636–25. doi: 10.1371/journal.ppat.1010636, PMID: 36240259 PMC9604950

[31] Trujillo M Cheung K Gao A Hoxie I Kannoly S Kubota N. Protocol for safe, affordable, and reproducible isolation and quantitation of SARS-CoV-2 RNA from wastewater. PLoS One. (2021) 16:e0257454–11. doi: 10.1371/journal.pone.0257454, PMID: 34555079 PMC8459947

[32] Greaney AJ Starr TN Barnes CO Weisblum Y Schmidt F Caskey M. Mapping mutations to the SARS-CoV-2 RBD that escape binding by different classes of antibodies. Nat Commun. (2021) 12:1–14., PMID: 34234131 10.1038/s41467-021-24435-8 PMC8263750

[33] Harvey WT Carabelli AM Jackson B Gupta RK Thomson EC Harrison EM. SARS-CoV-2 variants, spike mutations and immune escape. Nat Rev Microbiol. (2021) 19:409–24. doi: 10.1038/s41579-021-00573-0, PMID: 34075212 PMC8167834

[34] Liu H Wei P Kappler JW Marrack P Zhang G. SARS-CoV-2 variants of concern and variants of interest receptor binding domain mutations and virus infectivity. Front Immunol. (2022) 13:1–9. doi: 10.3389/fimmu.2022.825256, PMID: 35154144 PMC8828474

[35] Shang J Ye G Shi K Wan Y Luo C Aihara H. Structural basis of receptor recognition by SARS-CoV-2. Nature. (2020) 581:221–4. doi: 10.1038/s41586-020-2179-y, PMID: 32225175 PMC7328981

[36] Sun C Xie C Bu GL Zhong LY Zeng MS. Molecular characteristics, immune evasion, and impact of SARS-CoV-2 variants. Signal Transduct Target Ther. (2022) 7:202. doi: 10.1038/s41392-022-01039-2, PMID: 35764603 PMC9240077

[37] Cao Y Yisimayi A Jian F Song W Xiao T Wang L. BA.2.12.1, BA.4 and BA.5 escape antibodies elicited by omicron infection. Nature. (2022) 608:593–602. doi: 10.1038/s41586-022-04980-y, PMID: 35714668 PMC9385493

[38] Bate N Savva CG Moody PCE Brown EA Evans SE Ball JK. In vitro evolution predicts emerging SARS-CoV-2 mutations with high affinity for ACE2 and cross-species binding. PLoS Pathog. (2022) 18:e1010733–19. doi: 10.1371/journal.ppat.1010733, PMID: 35849637 PMC9333441

[39] Africa CDC (2023) Statement on the new COVID strain, EG.5 SARS-COV-2 subvariant. Available at: https://africacdc.org/news-item/statement-on-the-new-covid-strain-eg-5-sars-cov-2-subvariant/#:~:text=On%209%20August%202023%2C%20the,of%20E.G.5%20cases%20reported (Accessed October 01, 2023)

[40] Grant R Sacks JA Abraham P Chunsuttiwat S Cohen C Figueroa JP. When to update COVID-19 vaccine composition. Nat Med. (2023) 29:776–80. doi: 10.1038/s41591-023-02220-y, PMID: 36807683

[41] Iwu-Jaja C Ndlovu NL Rachida S Yousif M Taukobong S Macheke M. The role of wastewater-based epidemiology for SARS-CoV-2 in developing countries: cumulative evidence from South Africa supports sentinel site surveillance to guide public health decision-making. Sci Total Environ. (2023) 903:165817. doi: 10.1016/j.scitotenv.2023.165817, PMID: 37506905

[42] Tlhagale M Liphadzi S Bhagwan J Naidoo V Jonas K van Vuuren L. Establishment of local wastewater-based surveillance programmes in response to the spread and infection of COVID-19 – case studies from South Africa, the Netherlands, Turkey and England. J Water Health. (2022) 20:287–99. doi: 10.2166/WH.2022.185, PMID: 36366987

[43] Kim S Misra A. SNP genotyping: technologies and biomedical applications. Annu Rev Biomed Eng. (2007) 9:289–320. doi: 10.1146/annurev.bioeng.9.060906.152037 17391067

[44] Yu AT Hughes B Wolfe MK Leon T Duong D Rabe A. Estimating relative abundance of 2 SARS-CoV-2 variants through wastewater surveillance at 2 large metropolitan sites, United States. Emerg Infect Dis. (2022) 28:940–7. doi: 10.3201/eid2805.212488, PMID: 35349402 PMC9045426

[45] Lekana-Douki SE N'dilimabaka N Levasseur A Colson P Andeko JC Zong Minko O. Screening and whole genome sequencing of SARS-CoV-2 circulating during the first three waves of the COVID-19 pandemic in Libreville and the haut-Ogooué Province in Gabon. Front Med. (2022) 9:1–14. doi: 10.3389/fmed.2022.877391, PMID: 35655849 PMC9152426

