# Supplementary material for: Molecular epidemiology of SARS-CoV-2 in Northern South Africa: wastewater surveillance from January 2021 to May 2022
Source: Front Public Health. 2023 Dec 19;11:1309869. doi: 10.3389/fpubh.2023.1309869 (PMC10764116; doi:10.3389/fpubh.2023.1309869)
Supplement: Supplementary file 1 [file Table_1.DOCX]

**SUPPLEMENTARY MATERIAL**

**Unassembled reads of study sequences (57) submitted to NCBI SARS-CoV-2 SRA Database:** <https://www.ncbi.nlm.nih.gov/sra/PRJNA980445>

**Assembled reads of study sequences (3) submitted to NCBI GenBank Database:** <https://submit.ncbi.nlm.nih.gov/subs/?search=SUB13889355>

**Table 1:** PangoLIN and Nextclade assigned lineages and clades of study samples included in the analysis

| **No.** | **Sample Code** | **Date of Wastewater Sample Collection** | **Pangolin Lineage Designation & WHO Assignment** | **Nextclade Designation & WHO Assignment** | **Accession Number** |
| --- | --- | --- | --- | --- | --- |
| 1 | SI01W03I_21 | 18-Jan-21 | AY.45 (Delta) | 21J (Delta) | SRR24833901 |
| 2 | MA01W08I_21 | 22-Feb-21 | BA.2 (Omicron) | 21M (Omicron) | SRR25869424 |
| 3 | TH01W15I_21 | 12-Apr-21 | BA.1 (Omicron) | 21K (Omicron) | SRR25869423 |
| 4 | TH01W16I_21 | 19-Apr-21 | BA.1 (Omicron) | 21K (Omicron) | SRR25887293 |
| 5 | KA01W16I_21 | 19-Apr-21 | B.1.1.174 | 20B | SRR25869422 |
| 6 | TH01W18I_21 | 03-May-21 | BA.1 (Omicron) | 21K (Omicron) | SRR25916078 |
| 7 | TH01W19I_21 | 10-May-21 | B.1.1.174 | 20B | SRR25914800 |
| 8 | MA01W19I_21 | 10-May-21 | BA.2 (Omicron) | 21L (Omicron) | SRR25886446 |
| 9 | TZ01W19I_21 | 10-May-21 | AY.45 (Delta) | 21J (Delta) | SRR25869421 |
| 10 | SI01W19I_21 | 10-May-21 | AY.45 (Delta) | 21J (Delta) | SRR25887146 |
| 11 | LT01W19I_21 | 10-May-21 | B.1.1.174 | 20B | SRR25916080 |
| 12 | KA01W21I_21 | 24-May-21 | B.1.1 | 20B | SRR25914799 |
| 13 | KA01W22I_21 | 31-May-21 | BA.4 (Omicron) | 22A (Omicron) | SRR25914795 |
| 14 | LT01W25I_21 | 23-Jun-21 | B.1 | 20A | SRR25869420 |
| 15 | NK01W25I_21 | 23-Jun-21 | B.1.617.2 (Delta) | 21J (Delta) | SRR25886441 |
| 16 | KA01W26I_21 | 29-Jun-21 | B.1.1.174 | 20B | SRR25914798 |
| 17 | TZ01W26I_21 | 29-Jun-21 | BA.1 (Omicron) | 21K (Omicron) | SRR25887292 |
| 18 | TH01W26I_21 | 29-Jun-21 | B.1.1.174 | 20B | SRR25916079 |
| 19 | NK01W26I_21 | 29-Jun-21 | AY.38 (Delta) | 21J (Delta) | SRR25887148 |
| 20 | SI01W26I_21 | 29-Jun-21 | AY.45 (Delta) | 21J (Delta) | SRR25886439 |
| 21 | NK01W27I_21 | 05-Jul-21 | AY.45 (Delta) | 21J (Delta) | SRR25869419 |
| 22 | LT01W28I_21 | 13-Jul-21 | AY.45 (Delta) | 21J (Delta) | SRR25886442 |
| 23 | NK01W28I_21 | 13-Jul-21 | B.1.617.2 (Delta) | 20A | SRR25914797 |
| 24 | SI01W28I_21 | 13-Jul-21 | AY.45 (Delta) | 21J (Delta) | SRR25887153 |
| 25 | SI01W29I_21 | 19-Jul-21 | AY.45 (Delta) | 21J (Delta) | SRR25887299 |
| 26 | GI01W29I_21 | 19-Jul-21 | B.1.1 | 20B | SRR25869418 |
| 27 | KA01W29I_21 | 19-Jul-21 | B.1.351-like (Beta) | 20H (Beta) | SRR25886444 |
| 28 | TZ01W29I_21 | 19-Jul-21 | B.1.1.529 (Omicron) | 21K (Omicron) | SRR25887147 |
| 29 | TH01W30I_21 | 26-Jul-21 | B.1.617.2 (Delta) | 21J (Delta) | SRR25886445 |
| 30 | KA01W31I_21 | 02-Aug-21 | AY.45 (Delta) | 21J (Delta) | SRR25887156 |
| 31 | TH01W31I_21 | 02-Aug-21 | AY.45 (Delta) | 21J (Delta) | SRR25887151 |
| 32 | TZ01W31I_21 | 02-Aug-21 | BA.4.1 (Omicron) | 22A (Omicron) | SRR25886443 |
| 33 | NK01W31I_21 | 02-Aug-21 | AY.45 (Delta) | 21J (Delta) | SRR25887298 |
| 34 | KA01W32I_21 | 09-Aug-21 | B.1.617.2-like (Delta) | 21J (Delta) | SRR25887155 |
| 35 | SI01W32I_21 | 09-Aug-21 | BA.1 (Omicron) | 21K (Omicron) | SRR25869417 |
| 36 | KA01W33I_21 | 16-Aug-21 | B.1.617.2-like (Delta) | 21A (Delta) | SRR25887154 |
| 37 | TZ01W34I_21 | 23-Aug-21 | BE.1 (Omicron) | 22B (Omicron) | SRR25887149 |
| 38 | KA01W34I_21 | 23-Aug-21 | AY.45 (Delta) | 21J (Delta) | SRR25887297 |
| 39 | LT01W45I_21 | 08-Nov-21 | B.1.617.2 (Delta) | 21J (Delta) | SRR25887158 |
| 40 | SI01W46I_21 | 15-Nov-21 | B.1.1.529 (Omicron) | 21M (Omicron) | SRR25916025 |
| 41 | KA01W47I_21 | 22-Nov-21 | B.1.617.2 (Delta) | 21I (Delta) | SRR25887296 |
| 42 | TH01W49I_21 | 06-Dec-21 | AY.45 (Delta) | 21J (Delta) | SRR25887157 |
| 43 | LT01W49I_21 | 06-Dec-21 | B.1.1 | 20B | SRR25914796 |
| 44 | KA01W50I_21 | 13-Dec-21 | B.1.617.2 (Delta) | 21J (Delta) | SRR25916077 |
| 45 | TH01W50I_21 | 13-Dec-21 | B.1.1 | 20B | OR647513 |
| 46 | TZ01W50I_21 | 13-Dec-21 | B.1.1.529 (Omicron) | 20H (Beta) | OR647514 |
| 47 | LT01W50I_21 | 13-Dec-21 | BA.1 (Omicron) | 21K (Omicron) | OR647515 |
| 48 | TH01W51I_21 | 21-Dec-21 | AY.45 (Delta) | 21J (Delta) | SRR25887295 |
| 49 | TZ01W51I_21 | 21-Dec-21 | BA.4 (Omicron) | 22A (Omicron) | SRR25916024 |
| 50 | MA01W51I_21 | 21-Dec-21 | BA.2 (Omicron) | 21L (Omicron) | SRR25887152 |
| 51 | NK01W51I_21 | 21-Dec-21 | B.1.617.2 (Delta) | 21J (Delta) | SRR25914801 |
| 52 | KA01W02I_22 | 10-Jan-22 | B.1.351-like (Beta) | 20H (Beta) | SRR25887150 |
| 53 | NK01W03I_22 | 17-Jan-22 | BA.2 (Omicron) | 21L (Omicron) | SRR25916023 |
| 54 | NK01W04I_22 | 24-Jan-22 | BA.1 (Omicron) | 21K (Omicron) | SRR25916022 |
| 55 | SI01W05I_22 | 31-Jan-22 | AY.45 (Delta) | 21J (Delta) | SRR25887294 |
| 56 | SI01W17I_22 | 25-Apr-22 | AY.45 (Delta) | 21J (Delta) | SRR25914793 |
| 57 | GI01W18I_22 | 03-May-22 | BA.4 (Omicron) | 22A (Omicron) | SRR25886440 |
| 58 | TZ01W18I_22 | 02-May-22 | AY.45 (Delta) | 21J (Delta) | SRR25887291 |
| 59 | NK01W18I_22 | 02-May-22 | AY.45 (Delta) | 21J (Delta) | SRR25914794 |
| 60 | TZ01W19I_22 | 09-May-22 | BA.1 (Omicron) | 21K (Omicron) | SRR25916021 |
